# Superior Gluteal Nerve Anatomy and Its Injuries: Aiming for a More Secure Surgical Approach of the Pelvic Region

**DOI:** 10.3390/diagnostics13142314

**Published:** 2023-07-08

**Authors:** André R. Pinho, Maria J. Leite, João Lixa, Miguel R. Silva, Paula Vieira, João Nery-Monterroso, Mariana C. Bezerra, Hélio Alves, Maria Dulce Madeira, Pedro A. Pereira

**Affiliations:** 1 Unit of Anatomy, Department of Biomedicine, Faculty of Medicine, University of Porto, Alameda Professor Hernâni Monteiro, 4200-319 Porto, Portugal; arpcinco@hotmail.com (A.R.P.); up201906143@edu.med.up.pt (J.N.-M.); up201906038@edu.med.up.pt (M.C.B.); madeira@med.up.pt (M.D.M.); 2 Orthopaedics and Traumathology Department, Centro Hospitalar Universitário São João, Alameda Professor Hernâni Monteiro, 4200-319 Porto, Portugal; mjlcma@gmail.com (M.J.L.); joaolixa93@gmail.com (J.L.); mrelvas.silva@gmail.com (M.R.S.); paula.mpv@gmail.com (P.V.); 3 NeuroGen Research Group, Center for Health Technology and Services Research (CINTESIS), Rua Dr. Plácido da Costa, 4200-450 Porto, Portugal; 4 CINTESIS@RISE, Faculty of Medicine, University of Porto, Alameda Professor Hernâni Monteiro, 4200-319 Porto, Portugal

**Keywords:** superior gluteal nerve, greater sciatic notch, bony landmarks, cadaver study, dissection, surgical anatomy

## Abstract

Because most of the recognized causes of superior gluteal nerve (SGN) injury are iatrogenic, detailed knowledge of the anatomy of the SGN is crucial to prevent its injury associated with surgical procedures. This study aims to describe the precise location of SGN or its branches at the greater sciatic foramen, measure the distances of these neural structures to palpable bony landmarks, and evaluate the possible correlation between these parameters and pelvis size. Twenty human cadaveric hemipelvises were studied. After dissection to expose the SGN or its branches at the greater sciatic foramen, the distances from these neural structures to the greater trochanter (GT), to the anterior superior iliac spine (ASIS), to the posterior superior iliac spine (PSIS), to the ischial tuberosity (IT), and to the greater sciatic notch apex were measured. We found that at the greater sciatic foramen, the SGN emerges as a common trunk in 75% of hemipelvises, and already divided in its superior and inferior branches in 25% of hemipelvises. When the SGN exits the pelvis as a common trunk, it does so, in most cases, in contact with the bone at the apex of the greater sciatic notch or superior to the level of the apex. The median distance from the SGN at the greater sciatic notch to the PSIS, ASIS, GT and IT is 7.6 cm, 10.9 cm, 7.5 cm and 10.8 cm, respectively. We found a positive correlation between some of the analyzed parameters and the size of the pelvis. The anatomical data of this study may serve as pivotal guides during orthopedic pelvic surgery, contributing to minimize SNG iatrogenic lesions with significant implications in the patient’s quality of life.

## 1. Introduction

The superior gluteal nerve (SGN) is a branch of the sacral plexus that arises from the dorsal divisions of the fourth and fifth lumbar and first sacral ventral rami, and is the only neural structure to emerge at the gluteal region through the greater sciatic foramen superiorly to the piriformis muscle, in conjunction with the superior gluteal artery and vein [1,2,3,4,5,6,7,8]. The SGN divides into superior and inferior branches: the superior branch innervates the gluteus medius and occasionally the gluteus minimus muscles, the inferior branch innervates the gluteus medius and minimus, and ends in the tensor fasciae latae muscle [1,2,3,4,5,6,7,8]. The gluteus medius and minimus, acting from its proximal attachment, are the main abductors and medial rotators of the thigh, the latter function being performed by their anterior fibers. Acting from the femur, they play a critical role in maintaining the upright position of the trunk when the foot of the opposite side is raised from the ground during gait [8]. The actions of these muscles explain why lesions of the SGN may be responsible for gait abnormalities such as the Trendelenburg gait [8,9]. The tensor fasciae latae is a hip flexor and abductor. Its role in medial rotation, from the anatomical position, is minimal. This muscle helps to maintain upright posture while minimizing energy expenditure on muscle activity. In standing, it acts from below to steady the pelvis on the head of the femur and, through the iliotibial tract, helps to maintain the extended knee in a locked position. When standing on one limb, the tensor fasciae latae aids the gluteus medius in stabilizing the pelvis over the femur in the coronal plane [8]. 

The majority of proximal SGN injuries are iatrogenic and occur during surgery, with such lesions described during surgical approaches of the hip, acetabulum, pelvis and sacroiliac joints [10,11,12,13]. These lesions can occur due to traction of the adjacent structures during surgery, compression of the nerve or its vascular supply, improper retractor placement, or even direct neural transection, laceration, or thermic injury with the use of electrocautery or cement. In hip arthroplasty, the general incidence of nerve injury is approximately 1–4% [13]. Female sex and revision surgery are proven risk factors for iatrogenic surgical nerve injury [13]. Furthermore, during percutaneous fixation of the sacroiliac joint, a direct injury to the superior gluteal neurovascular bundle was described in up to 18% of the cases, related to screw positioning [11]. The most inferior branch of the SGN is most commonly injured during lateral and anterolateral approaches to the hip, corresponding to the injured nerve in 80% of the cases [13,14]. Regarding non-iatrogenic causes, SGN lesions are also described in acetabular fractures extending to the upper part of the greater sciatic notch (e.g., fractures of the posterior column) or fractures involving both columns, piriformis syndrome, pelvic fractures and, more rarely, with extrinsic compression from inflammatory or neoplastic masses [7,15]. Lesions of the SGN seem to occur more often than expected and there are very few studies regarding the injury of the SGN overall and the existing ones usually refer to the injury of its most inferior branch, as it reaches the tensor fasciae latae muscle [15].

To the best of our knowledge, there are no studies regarding the detailed position of the SGN or its branches, as it exits the greater sciatic foramen, nor its injury in that location. The goal of this study was to describe the relation of the SGN or its branches with the greater sciatic notch, and measure the distances between these neural structures at this location and selected bony references. Furthermore, we intended to evaluate if there was a correlation between the abovementioned parameters and the size of the pelvis. With this knowledge, we hope to contribute to the improved safety of surgical approaches of the region and eventually describe palpable anatomical references useful in open and percutaneous surgery around the hip and pelvis.

## 2. Materials and Methods

The cadavers used in this study derived from body donation with informed consent, written and signed by the donator himself (Portuguese Decree-law nº 274/99). As such, this anatomic study did not require investigational review board or ethics committee approval. Cadavers were received and embalmed at the Unit of Anatomy, Department of Biomedicine, Faculty of Medicine, University of Porto. Twenty hemipelvises from ten cadavers were selected from all formalin-embalmed full body adult cadavers dissected for this study. The remaining dissected cadavers were excluded based on the following criteria: surgical scars, evidence of previous trauma or surgery involving the hip joint and pelvis and/or altered normal anatomy by dissection procedures. The cadavers included in this study were all caucasian (6 males, 4 females). The age of the specimens ranged from 58 to 86 years (median of 78 years), while the height ranged from 1.49 to 1.75 m (median of 1.66 m). While in the female specimens the age ranged from 58 to 86 years (median of 64 years) and their height ranged from 1.49 to 1.68 m (median of 1.58 m), in male specimens age ranged from 75 to 85 years (median of 80 years) and height ranged from 1.64 to 1.75 m (median of 1.67 m). 

**Figure 1:**
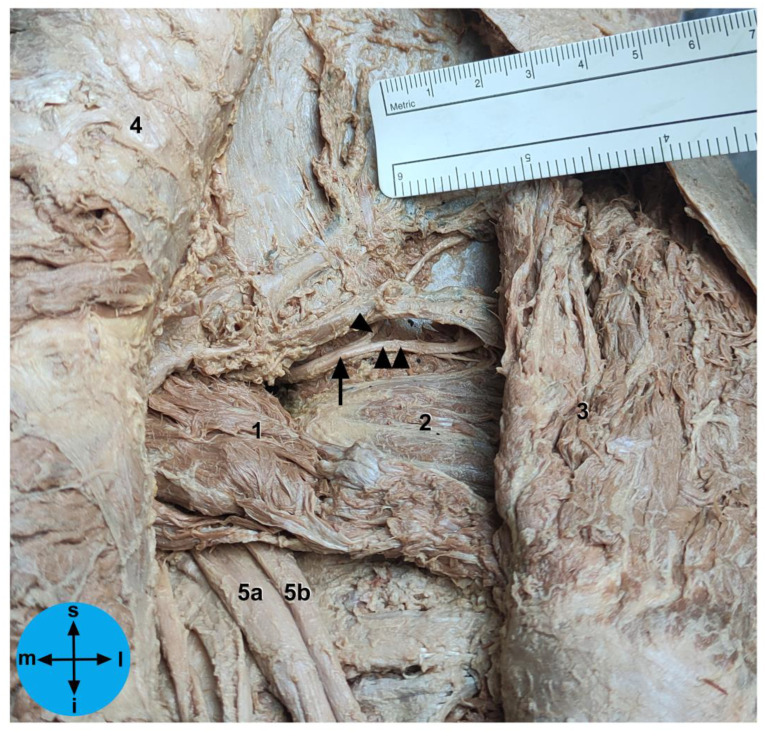
Posterior view of the gluteal region. The gluteus maximus muscle was reflected medially, and the gluteus medius and minimus muscles and the superior gluteal vessels were partially removed. The arrow indicates the superior gluteal nerve (SGN), the arrowhead indicates the superior branch of the SGN and the double arrowheads indicate the inferior branch of the SGN. Note that in this hemipelvis the sciatic nerve divides in the pelvis in the tibial and the common fibular nerves, both of which coursing below the piriformis muscle. 1: piriformis muscle; 2: gluteus minimus muscle; 3: gluteus medius muscle; 4: gluteus maximus muscle; 5a: tibial nerve; 5b: common fibular nerve; i: inferior; l: lateral; m: medial; s: superior.

**Figure 2:**
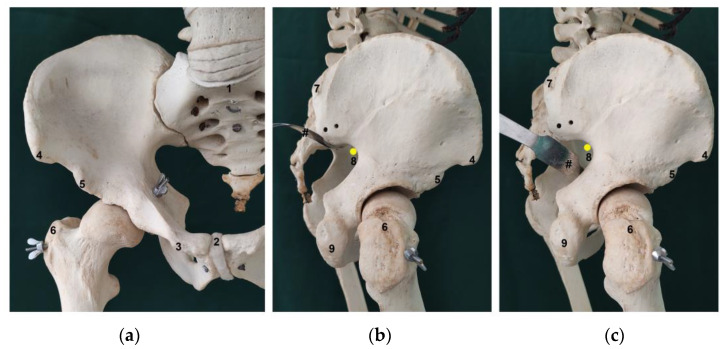
Anterior (**a**) and lateral (**b**,**c**) aspects of the skeletal pelvis and parts of the vertebral column and femur. The yellow solid circles represent the superior gluteal nerve (SGN), and the symbol # indicates an Hohmann retractor placed in the greater sciatic notch. 1: sacral promontory (SP); 2: pubic symphysis (PS); 3: pubic tubercle (PT); 4: anterior superior iliac spine (ASIS); 5: anterior inferior iliac spine (AIIS); 6: greater trochanter (GT); 7: posterior superior iliac spine (PSIS); 8: greater sciatic notch; 9: ischial tuberosity (IT).

The cadavers were routinely dissected in our Unit, and cadavers in which the trunk wall, abdominopelvic cavity and lower limb were preserved were considered for inclusion in the present study. As routine in our Unit, appropriate dissection techniques were performed by using proper dissection tools in order to achieve the teaching and research objectives of the human cadaveric dissection [16,17,18,19,20]. The specimens were carefully dissected in order not to disturb the normal anatomy of each region [8,20]. Regarding specifically the gluteal region (Figure 1), all dissection steps were based on those described previously in detail [20]. After dissection, the bony landmarks (Figure 2) were carefully identified and marked with a needle. A standardized measurement technique was developed, using a digital caliper and a standard surgical ruler, and all measurements were recorded by at least 2 different observers, over a period of 3 months and are expressed in centimeters (cm). Measurements were taken with the cadavers in the anatomical position, using four positions, i.e., supine, prone, and right and left lateral decubitus. Sex and laterality were also recorded. 

**Figure 3:**
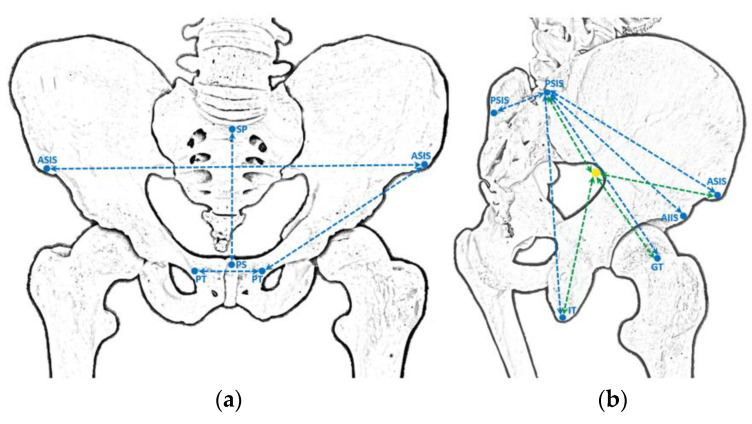
Schematic illustration of the measurements that were taken. Anterior (**a**) and posterolateral (**b**) aspects. The blue dashed lines indicate distances between bone structures, and the green dashed lines indicate distances between bone structures and the superior gluteal nerve (SGN, or its branches) at the level of the greater sciatic notch. The yellow solid circle represents the SGN. AIIS: anterior inferior iliac spine; ASIS: anterior superior iliac spine; GT: greater trochanter; IT: ischial tuberosity; PS: pubic symphysis; PSIS: posterior superior iliac spine; PT: pubic tubercle; SP: sacral promontory.

Several distances were evaluated to describe the relation between the SGN or its branches, the apex of the greater sciatic notch and several chosen palpable bony landmarks, as well as to elucidate the possible differences in these distances with specimen pelvis size. To obtain an estimate of the size of the pelvis we did several measurements, including: the 1) distance between the anterior superior and posterior superior iliac spines (ASIS-PSIS), the 2) distance between both anterior superior iliac spines (DASIS), the 3) distance between the anterior superior iliac spine and the pubic tubercle (ASIS-PT), the 4) distance between both pubic tubercles (DPT), the 5) distance between the midpoints of the sacral promontory and the upper border of the pubic symphysis (SP-PS), the 6) distance between both posterior superior iliac spines (DPSIS) and the 7) distance between the anterior inferior and posterior superior iliac spines (AIIS-PSIS). To elucidate the association between the pelvis and the other anatomical references, that could possibly be used to infer the exact location of the SGN or its branches at its exit from the pelvis, we also evaluated the distance between the PSIS and the ipsilateral 1) apex of the greater trochanter (GT) and 2) inferior part of the ischial tuberosity (IT) (Figure 3). 

**Figure 4:**
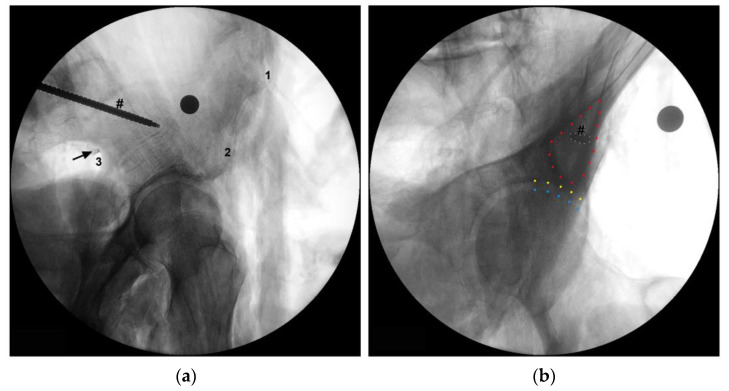
Fluoroscopic images of a simulation of an LC2 screw placement. (**a**) An iliac oblique view. (**b**) An obturator outlet view. The symbol # indicates the trocar that simulates the LC2 screw. (**a**) The arrow indicates the superior gluteal nerve (SGN) that was marked with a contrast medium at the point where it exits the pelvic cavity in direct contact with the greater sciatic notch. (**b**) The blue dashed line indicates part of the femoral head, and the yellow dashed line indicates part of the acetabulum. The area delimited by the red dashed line indicates the “teardrop” shaped bony channel above the greater sciatic notch within which the screw should be positioned. 1: anterior superior iliac spine (ASIS); 2: anterior inferior iliac spine (AIIS); 3: greater sciatic notch.

To take the measurements directly related to the SGN, as abovementioned, the gluteal region was carefully dissected in order to identify the superior gluteal neurovascular bundle above the piriformis muscle. Then, the neural structures of this bundle were carefully isolated at the level of the greater sciatic foramen without moving them from their proper position. Then, we carefully checked these neural structures at this level to identify the SGN or, in the hemipelvis in which the nerve divides before traversing the greater sciatic foramen, its branches (Figure 1). Then, we recorded the linear distances from the exit point of the SGN or its branches at the greater sciatic foramen to the: (1) apex of the GT, (2) PSIS, (3) ASIS and (4) the inferior part of the IT (Figure 3). We also determined the exact exit point of the SGN or its branches in the greater sciatic foramen (Figure 1). The emerging site of these neural structures from the pelvis in relation to the greater sciatic notch was recorded according to the following protocol: (a) the distance values were considered positive if the neural structures emerged superior (or dorsal) to the greater sciatic notch apex; (b) the distance values were considered negative if the neural structures emerged inferior to the greater sciatic notch apex. We also determined the linear distance of each neural structure to the bone at the greater sciatic notch. In one of the studied cadavers, we simulated the placement of an LC2 screw and we marked the SGN with a contrast medium at the point where it emerges from the pelvic cavity (Figure 4).

With regard to the statistical analysis, considering the skewness of the distributions of the pelvic parameters, descriptive statistics of the sample were performed using median, interquartile range (IQR), minimum and maximum values. The Mann–Whitney U test was used to assess possible differences regarding sex and sidedness. The association between the different pelvic measurements and the location of the SGN emergence from the greater sciatic foramen was evaluated through the Spearman’s correlation coefficient. The significance level was set at α = 0.05 and statistical analysis was performed using SPSS software (version 26, SPSS Inc., Chicago, IL, USA).

## 3. Results

As mentioned before, the sample is composed of 20 hemipelvises from 10 cadaveric specimens. Regarding the evaluated pelvic parameters representing the anteroposterior pelvic dimensions, the median distance between the anterior superior and posterior superior iliac spines (ASIS-PSIS) was 15.9 cm (min 13.5; max 17.7). Additionally, the distance between the sacral promontory and the upper border of the pubic symphysis (SP-PS) had a median value of 11.6 cm (min 10.5; max 12.2). Furthermore, the distance between the anterior inferior and posterior superior iliac spines (AIIS-PSIS) showed a median value of 15.7 cm (min 13.1; max 18.7) (Table 1). 

With respect to the transverse diameter of the pelvis, the measurements showed a median DPSIS of 9.2 cm (min 8.6; max 11.9). Furthermore, the DASIS had a median value of 22.6 cm (min 19.1; max 24.6). Additionally, the DPT had a median value of 5.2 cm (min 4.5; max 5.6). Representing the vertical diameter of the pelvis, the ASIS-PT distance showed a median value of 12.3 cm (min 10.2; max 13.9) (Table 1). In our sample, there were no statistically significant differences in the measured pelvic parameters regarding side or sex, even though the anteroposterior diameter of the pelvic inlet (measured between the midpoints of the sacral promontory and upper border of the pubic symphysis) was close to being significantly different between sexes (median value of 11.8 cm in females and 11.2 cm in male cadavers; *p* = 0.067).

In regard to the chosen anatomical references that could possibly be used to infer the exact location of the SGN or its branches at its exit from the pelvis, the distances between the PSIS and the ipsilateral 1) GT and the 2) IT showed a median of 14.4 cm (min 11.1; max 16.3; IQR 2.9) and 16.9 cm (min 13.0; max 18.6; IQR 2.2). 

Regarding the SGN, in all specimens included in this study, we found the SGN exiting the pelvic cavity through the greater sciatic foramen above the piriformis muscle. At the greater sciatic notch, the SGN emerges as a single branch in 15 hemipelvises (75%). In 5 hemipelvises (25%) the SGN emerges already divided in its two branches. In regard to the subgroup of hemipelvises in which the SGN emerges as a single branch, in the vast majority of cases (10 of the 15 hemipelvises) the nerve emerged in direct contact with the bone at the apex of the greater sciatic notch (median distance of 0.0 cm). In 4 hemipelvises the SNG emerged superiorly to the greater sciatic notch apex, distancing between 0.1 to 0.5 cm. In one hemipelvis the SGN emerged inferiorly to the greater sciatic notch apex. The median distance from the exit of the SGN at the greater sciatic notch were 7.6 cm to the PSIS (min 7.2; max 8.4), 10.9 cm to the ASIS (min 9.9; max 11.8), 7,5 cm to the apex of the GT (min 5.5; max 9.4) and 10.8 cm to the inferior part of the IT (min 8.8; max 12.1) (Table 2). 

Concerning the 5 hemipelvises in which the SGN emerged already dived in its superior and inferior branches, each of its branches were characterized separately. The superior branch emerged in all cases superior to the greater sciatic notch apex, with a median distance of 0.3 cm (min 0.1; max 1.0). The superior branch of the SGN was located at a median distance of 6.6 cm from the PSIS (min 6.2; max 7.0), 11,0 cm from the ASIS (min 9.5; max 11.3), 8.5 cm from the GT (min 6.7; max 9.0) and 10.7 cm from the IT (min 7.9; max 11.4). The inferior branch of the SGN emerged in all cases at the apex (1 hemipelvis) or inferior to the greater sciatic notch apex (4 hemipelvises), with a median distance of 0.2 cm from that reference (min −0.5; max 0.0). This branch was in closer relation with the GT (median distance of 8.1 cm; min 6.4; max 8.7), the IT (median distance of 10.5 cm; min 7.7; max 11.1) and the ASIS (median distance of 10.5 cm; min 9.2; max 10.9), but more distant from the PSIS (median distance 7.4 cm; min 6.6; max 7.8) (Table 2). There were no statistically significant differences according to side or sex regarding the distance between the SGN and the chosen pelvic bony anatomical references, although the distance to the apex of the GT showed a tendency to be inferior in females (median distance of 7.9 vs. 6.5 cm, *p* = 0.069). 

Regarding the relation between the size of the pelvis and the distance from the SGN to the chosen bone structures, our data showed that pelvises with greater anteroposterior diameters (greater ASIS to PSIS distance) were associated with smaller distances from the SGN to the apex of the greater sciatic notch, either for the nerves that emerged as a common trunk or already divided in its branches. This association showed a modest negative correlation of −0.48 (SGN and superior branch) or −0.45 (SGN and inferior branch). There was also a significant association between greater pelvic diameter (ASIS-PSIS distance) and greater distance from the SGN to the inferior part of the IT, including hemipelvises in which the SGN emerged as a common trunk, as well as for both branches of the SGN that emerged already divided (*p* = 0.025 and *p* = 0.022, respectively). This association showed a modest positive correlation, with a correlation coefficient around 0.50 for both SGN branches (Table 3). 

Concerning the relationship between the SGN emerging site and the remaining pelvic parameters, most of the chosen parameters did not show a significant association with the distance from the SGN to the IT, GT or greater sciatic notch, including the (1) DASIS, (2) ASIS-PT distance, (3) DPT, (4) SP-PS distance, (5) DPSIS and (6) the AIIS-PSIS distance.

On the other hand, we found statistically significant associations between the distance from the PSIS to the GT and the distance from the SGN to the IT and to the GT (*p* < 0.001), showing strong positive correlations with correlation coefficients ≥0.75. Furthermore, the distance between the PSIS and the IT also showed a significant association with the emerging SGN site and its distance to the IT, including the specimens with SGN emerging as a common trunk and both the superior and inferior branches (*p* < 0.001). This relation showed a strong positive correlation (correlation coefficient of 0.78 and 0.77, respectively). The distance between the PSIS and the IT also showed a statistically significant association with the distance from the GT to the SGN emerging site, including the SGN trunk and the superior (*p* = 0.018) or the inferior (*p* = 0.012) branches, with a weak to moderate correlation (correlation coefficient of 0.52 and 0.55, respectively) (Table 3).

## 4. Discussion

The main goal of this study was to define the precise exiting point of the SGN or its branches at the greater sciatic foramen, regarding various bone structures. Some of the anatomic references were chosen due to their easy accessibility for superficial palpation by a physician, and their clinical importance with regard to the surgical anatomy of the region. 

A sample of 20 hemipelvises was used to evaluate the pelvic morphology, the relation of the SGN with the greater sciatic notch, the distance from the SGN or its branches at the point they leave the pelvic cavity to the chosen bony anatomical landmarks. After the anatomical and morphological evaluation, our sample data were evaluated, in the search for associations between these parameters and differences according to laterality and sex. 

Regarding laterality, no differences were found in any of the parameters evaluated and this can help exclude any role of a functional dominant limb. Additionally, there were no significant differences when the two sexes were compared. However, the absence of statistically significant differences may be due to the small number of specimens. Indeed, the sexual differences in the pelvis are well known and widely described, and are unavoidably linked to function [8]. For instance, in our sample, the anteroposterior diameter (true conjugate) of the pelvic inlet (superior pelvic aperture), measured between the midpoints of the sacral promontory and upper border of the pubic symphysis, had a median value of 11.8 cm in female and 11.2 cm in male cadavers. These results show a tendency towards a greater anteroposterior diameter of the pelvic inlet in females, in line with those stated in classical textbooks, where it is stated that, on average, this diameter is 11.2 cm in adult females and 10.0 cm in adult males [8].

In 75% of the hemipelvises, the SGN emerged as a common trunk through the greater sciatic foramen, and in 25% of cases, it emerged already divided. Concerning the 15 cases in which the SGN emerged as a single branch, 10 of them had the nerve in direct contact with the bone at the apex of the greater sciatic notch. In our sample, this is the location of greater risk of possible iatrogenic lesion to the SGN. According to our sample data, the most secure location for surgical exploration and for a surgical retractor placement, seems to be inferior to the apex of the greater sciatic notch when we approach the hip or near the posterior inferior iliac spine (PIIS) when taking a posterior approach to the sacroiliac joint or to the fixation of the crescent iliac fracture. 

In our sample, only 25% of hemipelvises had a SGN emerging already divided in its superior and inferior branches at the greater sciatic notch. However, in hemipelvises in which the SGN emerges already divided, care should be taken not to injure the inferior branch, which emerged in every case at or inferior to the greater sciatic notch apex, and in closer relation to the GT (median distance of 8.1 cm) and the IT (median distance of 10.5 cm) than the superior branch. There were no significant differences according to laterality. Furthermore, although no significant differences were recorded according to sex, the distance between the SGN and the apex of the GT showed a trend towards being smaller in females (*p* = 0.069), possibly due to the generally smaller stature of females compared to males, which is corroborated by the data from our sample. This can contribute to the established fact that female patients have greater risk for iatrogenic nerve injury in hip surgery [13]. This difference is probably due to their lesser height, resulting in smaller distances between bony landmarks and neural structures and consequently, in a higher risk of injury. The lower soft tissue mass present in females is also suggested to be an important factor in this higher risk [21]. Although no significant differences were found between sex, the authors feel it is important to remain attentive, especially in females, when surgically approaching the trochanteric area, namely when choosing the position of Hohmann retractors that can possibly cause entrapment of the SGN distally to the greater sciatic notch. Picado et al. [22] evaluated 40 patients subjected to total hip arthroplasty using the direct lateral approach for nerve injury using electromyography 4 weeks post-operatively. Injury to the SGN was found in 17 patients, and although most of these were transient, injury to the SGN can result in significant morbidity such as Trendelenburg gait [22]. In our study, the SGN showed great proximity to the GT, with a median distance of 7.5 cm from its apex. Several studies have evaluated the distance between the apex of the GT and the inferior branch of the SGN, in order to describe a safe zone for the lateral/anterolateral approach to the hip [22,23,24,25]. Although results vary, it was possible to define a safe zone, limiting the incision between 3–7 cm from the apex of the GT cranially. It has been proposed that if this limit is exceeded, the neurovascular bundle is at risk of lesion development [22,23,24,25]. Ray et al. [23] studied the branching pattern and length of the SGN, from its exit in the greater sciatic foramen to the point where it pierces the glutei (medius and minimus) and the tensor fasciae latae muscles [23]. The mean distance from the apex of the GT to its emergence was 7.26 ± 1.65 cm. These findings are in close range to ours, since in our study, the median distance from the apex of the GT to the SGN emergence was 7.5 cm. 

The SGN is also in close proximity to the greater sciatic notch which is frequently in contact with retractors used in hip arthroplasty for acetabulum exposure or when approaching the dorsal portion of the iliac bone for fixation of the crescent iliac fragment or sacroiliac joint (Figure 2b,c). In our study, we found that the SGN emerged, in most cases, in direct contact with the bone at the apex of the greater sciatic notch. This places the SGN in the anterosuperior quadrant of the greater sciatic notch, and thus in close contact with the acetabular posterior wall/column. It is the authors recommendation to exercise caution when placing Hohmann retractors in the posterosuperior and superior region of the acetabular wall, due to the increased risk of entrapment of the SGN between the retractor and the bone at the greater sciatic notch. The same recommendation is mandatory for the posterior sacroiliac approach when we do an open reduction of the iliac fragment or sacroiliac joint and we need to stay in the safe zone in the proximity of PIIS or the first 7.6 cm of the superior border of greater sciatic notch. This relation between the SGN and the pelvic structures is particularly relevant in smaller patients. As would be expected, patients with smaller distances from the PSIS to the GT or IT had the SGN in closer relation with the GT and IT, and were potentially at higher risk of iatrogenic injury. 

There are also reports of injury to the SGN during percutaneous iliosacral screw insertion. Collinge et al. [11] performed a study in which the 58 sacroiliac screws were placed in the first sacral bodies, and lesions of the superior branch of the SGN and superior gluteal vessels were observed in 10 of the 58 (18%) [11]. In this context, it is important to highlight that two of the bone structures whose distances to the SGN or its branches at the greater sciatic notch were taken, i.e., the ASIS and the GT, are used as anatomic landmarks for percutaneous iliosacral screw fixation [26]. In our study, besides the GT, the SGN was also in close proximity to the PSIS, at a distance of 7.6 cm from it. The PSIS is an important surgical reference, due to its superficial location and usefulness in the localization of PIIS, making it a useful guide for percutaneous fixation of crescent ilium fractures (LC2 Screw). Therefore, we recommend caution when placing these screws, as a screw directed at the AIIS with too inferior an orientation can induce fracture of the upper limit of the greater sciatic notch and concomitant lesion of the SGN or its branches taking into account the close relation between these neural structures and the greater sciatic notch (Figure 4). 

Regarding the IT, this is a safe starting point to aim retrograde posterior column screws during pelvic percutaneous fixation, distancing in median 10.8 cm from the SGN. However, care should be taken not to direct the screw too posteriorly when trying to avoid the hip joint, due to the risk of entering the greater sciatic notch and injuring the adjacent structures. 

Although our study has produced important surgical and clinical anatomical findings, which we hope can influence surgical practice, it also has some limitations that are generally observed in studies that are performed with cadavers. Nevertheless, we attempted to minimize these limitations. The main limitation is related to the changes in the volume and trophicity of muscle mass with death and fixation techniques. We tried to minimize this limitation by using exclusively bony references. Furthermore, we measured some reference parameters, and the obtained results were similar to data previously reported in the literature, which unequivocally provides robustness to our results. Furthermore, due to availability and cost, the number of cadaver specimens is relatively reduced, possibly contributing to the fact that some of our results could not achieve statistical significance. Finally, another limitation of our study is the absence of clinical data related to the studied cadavers which prevented the establishment of any correlation between anatomical and clinical aspects.

## 5. Conclusions

It is of paramount importance to recognize that when SGN exits the pelvis through the greater sciatic foramen as a common trunk, it does so, in most cases, in close contact with the bone at the apex of the greater sciatic notch or superior to the level of the apex. It is the view of the authors that although there is a safe distance to perform surgery around the hip or posterior sacroiliac approach without isolating the nerve, care should be taken when placing retractors around the greater sciatic notch. Besides that, in the surgical approach during hip arthroplasty, the SGN showed greater proximity to the GT, and surgeons should be aware of this relation, especially in smaller patients in which this distance is significantly less. Regarding percutaneous fixation, these techniques seem to be relatively safe; however, depending on the entry point, one should pay close attention to the screw trajectory to avoid any potential iatrogenic lesions of the SGN.

## Figures and Tables

**Table 1:** Description of pelvic morphology (distances in centimeters).

	Anteroposterior Pelvic Dimensions			Transverse Pelvic Dimensions			Vertical Pelvic Dimensions
	ASIS-PSIS	SP-PS	AIIS-PSIS	DPSIS	DASIS	DPT	ASIS-PT
Median (IQR)	15.9 (1.4)	11.6 (1.0)	15.7 (2.7)	9.2 (0.8)	22.6 (1.7)	5.2 (0.5)	12.3 (1.7)
Minimum	13.5	10.5	13.1	8.6	19.1	4.5	10.2
Maximum	17.7	12.2	18.7	11.9	24.6	5.6	13.9

ASIS—anterior superior iliac spine, AIIS—anterior inferior iliac spine, DASIS—distance between both anterior superior iliac spines, DPSIS—distance between both posterior superior iliac spines, DPT—distance between both pubic tubercles, PS—pubic symphysis, PSIS—posterior superior iliac spine, PT—pubic tubercle, SP—sacral promontory.

**Table 2:** Description of SGN (or branches) at the greater sciatic foramen exit location and defined bony landmarks (distances in centimeters).

	Greater Sciatic Notch *	PSIS	ASIS	GT	IT
SGN Common trunk (*n* = 15)					
Median (IQR)	0.0 (0.1)	7.6 (0.8)	10.9 (0.6)	7.5 (2.1)	10.8 (1.6)
Minimum	−0.1	7.2	9.9	5.5	8.8
Maximum	0.5	8.4	11.8	9.4	12.1
SGN superior branch (*n* = 5)					
Median (IQR)	0.3 (0.6)	6.6 (0.8)	11.0 (1.6)	8.5 (2.0)	10.7 (3.1)
Minimum	0.1	6.2	9.5	6.7	7.9
Maximum	1.0	7.0	11.3	9.0	11.4
SGN inferior branch (*n* = 5)					
Median (IQR)	−0.2 (0.4)	7.4 (1.3)	10.5 (1.5)	8.1 (1.8)	10.5 (2.9)
Minimum	−0.5	6.6	9.2	6.4	7.7
Maximum	0.0	7.8	10.9	8.7	11.1

ASIS—anterior superior iliac spine, GT—greater trochanter, IQR – interquartile range, IT—ischial tuberosity, PSIS—posterior superior iliac spine, SGN – superior gluteal nerve. * by convention, negative values report to neural structures emerging inferior to the apex of the greater sciatic notch.

**Table 3:** Correlation between SGN (or branches) distance to defined bony landmarks and pelvic dimensions.

	ASIS-PSIS		PSIS-GT		PSIS-IT	
	ρ	*p*-Value	ρ	*p*-Value	ρ	*p*-Value
SGN CT or SB (*n* = 20)						
Absolute distance to the apex of greater sciatic notch	−0.48	0.034 *	−0.19	0.435	−0.25	0.282
Distance to GT	0.32	0.169	0.78	<0.001 *	0.52	0.018 *
Distance to IT	0.50	0.025 *	0.77	<0.001 *	0.78	<0.001 *
SGN CT or IB (*n* = 20)						
Absolute distance to the apex of greater sciatic notch	−0.45	0.046 *	−0.06	0.788	−0.06	0.795
Distance to GT	0.37	0.110	0.80	<0.001 *	0.55	0.012 *
Distance to IT	0.51	0.022 *	0.75	<0.001 *	0.77	<0.001 *

ASIS—anterior superior iliac spine, CT—common trunk, IB—inferior branch, IT—ischial tuberosity, GT—greater trochanter, PSIS—posterior superior iliac spine, SB—superior branch, SGN—superior gluteal nerve, ρ—Spearman’s rho correlation coefficient, *—*p*-value <0.05.

## Data Availability

Data are available upon reasonable request to the corresponding author.

## Abbreviations

The following abbreviations are used in this manuscript:

AIIS	anterior inferior iliac spine
AIIS-PSIS	distance between the anterior inferior and posterior superior iliac spine
ASIS	anterior superior iliac spine
ASIS-PSIS	distance between the anterior superior and posterior superior iliac spine
ASIS-PT	distance between the anterior superior iliac spine and the pubic tubercle
CT	common trunk
DASIS	distance between both the anterior and superior iliac spine
DPSIS	distance between both the posterior and superior iliac spine
DPT	distance between both pubic tubercles
GT	greater trochanter
IB	inferior branch
IQR	interquartile range
IT	ischial tuberosity
PIIS	posterior inferior iliac spine
PS	pubic symphysis
PSIS	posterior superior iliac spine
PT	pubic tubercle
SB	superior branch
SGN	superior gluteal nerve
SP	sacral promontory
SP-PS	distance between the midpoints of the sacral promontory and the upper border of the pubic symphysis
